# Sydenham’s Chorea as the First Manifestation of Rheumatic Fever in Two Boys

**DOI:** 10.31138/mjr.32.4.369

**Published:** 2021-12-27

**Authors:** Jozélio Freire de Carvalho, Leonid P. Churilov

**Affiliations:** 1 Institute for Health Sciences from Federal University of Bahia, Salvador, Bahia, Brazil,; 2 Laboratory of the Mosaic of Autoimmunity, Saint Petersburg State University, Russia

## Abstract

Sydenham’s chorea is a cardinal manifestation of rheumatic fever, but it is more common in girls. We describe two boys who developed Sydenham’s chorea and were successfully treated.

**Case 1:**

A 5-year-old boy started an involuntary movement of his hand, progressing to all the upper limbs, bilaterally. The family observed a deterioration in his handwriting skills. Heart auscultation did not reveal any murmur, and the oropharynx examination was normal. A brain magnetic resonance imaging, cerebrospinal fluid, and echocardiography were interpreted as normal. He was treated with valproate 2.5mL twice a day. Prophylaxis with benzathine penicillin was started using 600,000IU every 21 days. After four months, the patient was asymptomatic, and valproate was tapered off.

**Case 2:**

A 7-year-old boy with a long history of frequent otitis and pharyngitis started involuntary movements of his face and on his upper limbs, and also, his mother noted tics on his face. A brain magnetic resonance imaging and transthoracic echocardiography were normal. He was treated with haloperidol 10 drops (1mg) twice a day, and prophylaxis with benzathine penicillin was started using 600,000IU every 21 days. After three months, the patient was asymptomatic, all chorea manifestations resolved, haloperidol was then tapered off. In conclusion, this study illustrates two uncommon cases of boys who developed Sydenham’s chorea and had a good outcome.

## INTRODUCTION

Rheumatic fever (RF) or Bouillaud–Sokol’skiy disease is an immunopathological illness caused by Streptococcus A-beta-haemolytic group, following a tonsillitis process. Its first medical descriptions belong to a French physician Jean-Baptiste Bouillaud (in 1832 as a joint disease) and a Russian internist Grigory I. Sokol’skiy (in 1838 as a heart disease).^1^ Its pathogenesis combines immune complex hypersensitivity reactions involving endothelium and autoimmune processes caused by molecular mimicry of streptococcal M5 protein peptides and self-antigens.^2^ Two or more weeks after the streptococcal infection, the children initiate migratory polyarthritis and, in some cases, endocarditis, with more rare involvement of other cardiac layers. Some manifestations have a late-onset such as erythema migrans and Sydenham’s chorea (SC).^3^ The SC was first described by German physician Gregor Horst in 1625, but the eponym was given after British physician Thomas Sydenham, who later (1686) detailed its description.^1,4^

In SC autoantibodies derived due to antigen mimicry target the cerebral basal ganglia brain cells (in corpus striatum and caudate nucleus), thus altering the balance between dopaminergic, and inhibitory gamma-aminobutyric acid (GABA) systems.^5^ It is not purely rheumatic manifestation, sometimes, although seldom, occurring in other autoimmune diseases (see below).

In fact, SC has a late appearance, 6–8 weeks or even several months after the streptococcal infection. The children, usually girls, initiate involuntary movement of their limbs that improve with rest or sleep, and worse with anxiety or with the intention of improvement.^3,5^

This article aims to report two boys who developed SC as the first presentation of RF.

## CASE REPORT

### CASE 1

A 5-year-old boy with a negative past medical history started in August 2014, involuntary movements of his left hand and then the right one, progressing to all the upper limbs, bilaterally. The family observed a deterioration in his handwriting skills. He was referred to a paediatric neurologist who performed electroencephalography that was normal, and the doctor gave him the diagnosis of chorea. He was treated with valproate 2.5mL (125 mg) twice a day, and then he was referred to us. His physical examination showed that he twisted his hands and had frequent jerking movements. The heart was normal, and no nodules or erythema were observed in his skin. Laboratory tests demonstrated antistreptolysin O (ASLO) titre of 80 IU/mL (normal range: < 250 IU/mL), negative oropharynx culture, C-reactive protein of 0.03mg/L, erythrocyte sedimentation rate of 4mm/1^st^ hour, normal levels of acid alpha1-glycoprotein and normal serum electrophoresis. Positive antinuclear antibodies with a titre of 1:640, dense fine speckled pattern. Anti-dsDNA, anti-ribosomal P, anti-Ro/SS-A, anti-La/SS-B, anti-U1RNP, anti-Sm, ANCA, anti-CCP, anti-thyroglobulin, anti-thyroperoxidase, rheumatoid factor, IgG and IgM anticardiolipin and lupus anticoagulant were all negative. Complement levels were normal. Vitamin B12 was 813 pg/mL,25-OH-vitamin D of 42 ng/mL, increased TSH of 6.2 mU/mL (nr: 0.3–4.0 mU/L), and normal free T4 0.99 ng/dL (nr: 0.9–1.8 ng/dL). Serology for syphilis, HIV 1 and 2, and HTLV I and II were negative. A magnetic resonance imaging of the brain was normal. A transthoracic echocardiography was interpreted as normal. A cerebrospinal fluid study was completely normal. A prophylaxis with benzathine penicillin was started using 600,000IU every 21 days. His weight was 22.5kg. After four months, the patient was asymptomatic, valproate was tapered off, and ASLO was < 25 units. TSH increased to 12.5 mcU/mL, and levothyroxine 25mcg/day was initiated. Currently, he is 11 years old, asymptomatic, and under prophylaxis.

### CASE 2

A 7-year-old boy with a long history of frequent otitis and pharyngitis, started involuntary movements of his face and on his upper limbs in February 2012, and his mother noted tics on his face (closing eyes several times) and repeated movements of his hands and arms. Interestingly, his mother and his grandparents had a history of RF. The physical examination showed that he twisted his hands and arms, and he had frequent jerking movements; tics on his face (closing eyes) were confirmed, no abnormal heart murmur was observed, oropharynx was normal, and no nodules or erythema were observed. Laboratory testes demonstrated antistreptolysin O titre of 21.8 IU/mL (normal range: < 250 IU/mL), negative oropharynx culture, and C-reactive protein 0.1mg/L, erythrocyte sedimentation rate of 2 mm/1^st^ hour, normal levels of acid alpha1-glycoprotein, and normal serum electrophoresis. Antinuclear antibodies, anti-dsDNA, anti-ribosomal P, rheumatoid factor, IgG and IgM anticardiolipin, and lupus anticoagulant were all negative. Complement levels were normal. A magnetic resonance imaging of the brain and transthoracic echocardiography were interpreted as normal. He was treated with haloperidol 10 drops (1mg) twice a day. A prophylaxis with benzathine penicillin was started using 600,000IU every 21 days. His weight was 24kg. After three months, the patient was asymptomatic, all chorea manifestations resolved, haloperidol was then tapered off, and ASLO was < 25 units. Currently, he is 15 years old, asymptomatic, and under prophylaxis with penicillin.

## DISCUSSION

The present article case report adds two new cases of Sydenham’s chorea in boys with excellent outcomes. There are several causes for chorea, including atypical seizures, brain tumours, stroke, drug intoxication, carbon monoxide poisoning, hyperthyroidism, hypoglycaemia, hereditary disorders such as Huntington’s chorea, Wilson’s disease, pregnancy, viral encephalitis, and autoimmune choreas.^6,7^ The last is a group of diseases with an autoimmune pathogenesis causing this neurological syndrome. It includes rheumatic SC and autoimmune choreas associated with systemic lupus erythematosus, primary antiphospholipid antibody syndrome (APS), Behçet disease, paraneoplastic, and anti-*N*-methyl-d-aspartate receptor encephalitis.^6^ Interestingly, our group has described a case series of four patients with chorea in 89 primary APS and concluded that 4.5% of primary APS individuals had chorea, predominately before the APS diagnosis, and characteristically this neurological abnormality was associated with rheumatic fever and thrombocytopenia.^8^ In our patients, lupus and the antiphospholipid syndrome were excluded since no clinical or laboratory features of these disorders and autoantibodies associated with them were found. Besides, the patients were children, and no tumour was thought or found in them to suppose in paraneoplastic chorea. Moreover, finally, no evidence of encephalitis was presented by the patients.

SC is the most common cause of chorea in childhood, and it is a cardinal feature of rheumatic fever. That is why its presence alone is enough to make the RF diagnosis.^3^ Usually, the SC onset is delayed, varying from 1 to 6 months of the streptococcal infections. SC is a disease of childhood or early adolescence, mainly between 5 to 15 years old, and its incidence varies from 1% to 15% depending on the geographical region analysed.^3^ Prior to age of 5 years SC in RF is extremely uncommon.^9^ Commonly it is bilateral, and the girls are more affected than boys in a ratio of 2–3:1.^5,10^ However, there are some descriptions of boys with SC, and original text by T. Sydenham just described a case of boy.^4^

One reasonable hypothesis is that genetically prone children are exposed to streptococcal infection, so antibodies attack cells in the basal ganglia and cause inflammation due to a molecular mimicry mechanism.^5,11^ Antibodies to N-acetyl-beta-D-glucosamine, an immunodominant epitope of a streptococcal carbohydrate and one principal constituent of the bacterial cell wall, were isolated from a patient with SC and, these antibodies were subsequently shown to react with gangliosides expressed in the human brain.^12^ Furthermore, SC patients present autoantibodies against neurons of the caudate and subthalamic nuclei of the basal ganglia in an analysis using immunohistochemistry and post-mortem brain tissue. Additional investigations have revealed high rates of neuronal reactive antibodies in the serum of children with SC.^13^

Symptomatic treatment of SC has the objective of minimizing symptom severity and shortening the course of illness. Dopaminergic medications are thought to help reduce the frequency and amplitude of the choreoathetosis movements. However, they carry significant side effects. The glucocorticoids are usually employed to treat acute SC, and more recently, plasmapheresis and intravenous immunoglobulin have shown efficacy in its treatment of SC.^14^ The primary goal in the SC approach is the prophylaxis of streptococcal infection driven by antibiotics. Our patients received valproate and haloperidol and had an excellent symptomatic neurological control, and more importantly, the benzathine penicillin blocked any other recurrence of RF.

## CONCLUSION

In conclusion, this article describes Sydenham’s chorea in two boys. It alerts to an excellent outcome when its diagnosis is performed, and symptomatic treatment and effective prophylaxis are prompt initiated.

## ABBREVIATIONS

ASLO: Antistreptolysin
GABA: Gamma-aminobutyric acid
RF: Rheumatic fever
SC: Sydenham’s chorea
